# Supplementary material for: The Effects of Action Observation Therapy as a Rehabilitation Tool in Parkinson’s Disease Patients: A Systematic Review
Source: Int J Environ Res Public Health. 2022 Mar 11;19(6):3311. doi: 10.3390/ijerph19063311 (PMC8949895; doi:10.3390/ijerph19063311)
Supplement: Supplementary file 1 [file ijerph-19-03311-s001.zip › ijerph-1612730-supplementary.pdf]

Search Strategy

PubMed: ((((("Parkinson disease") OR ("Parkinson's disease")) OR (Parkinson*)) OR (PD)) OR (Parkinsonism)) AND (((((((("Action observation") OR ("Action observation training")) OR ("Action observation treatment")) OR ("Action observation therapy")) OR ("Action observation-execution")) OR ("Motor observation")) OR ("Movement observation")) OR ("Motion observation")) OR ("Gesture observation"))

Science Direct: ("Parkinson disease" OR "Parkinson's disease" OR Parkinson OR PD OR Parkinsonism) * Find articles with these terms
("Action observation" OR "Action observation training" OR "Action observation treatment" OR "Action observation therapy" OR "Action observation-execution" OR "Motor observation" OR "Movement observation" OR "Motion observation" OR "Gesture observation") * Title, abstract or author-specified keywords

Cochrane Library: ("Parkinson disease" OR "Parkinson's disease" OR Parkinson* OR PD OR Parkinsonism) AND ("Action observation" OR "Action observation training" OR "Action observation treatment" OR "Action observation therapy" OR "Action observation-execution" OR "Motor observation" OR "Movement observation" OR "Motion observation" OR "Gesture observation") * in Title - Abstract - Keyword

Scopus: TITLE-ABS-KEY("Parkinson disease") OR TITLE-ABS-KEY("Parkinson's disease") OR TITLE-ABS-KEY(Parkinson*) OR TITLE-ABS-KEY(PD) OR TITLE-ABS-KEY(Parkinsonism) AND TITLE-ABS-KEY("Action observation") OR TITLE-ABS-KEY("Action observation training") OR TITLE-ABS-KEY("Action observation treatment") OR TITLE-ABS-KEY("Action observation therapy") OR TITLE-ABS-KEY("Action observation-execution") OR TITLE-ABS-KEY("Motor observation") OR TITLE-ABS-KEY("Movement observation") OR TITLE-ABS-KEY("Motion observation") OR TITLE-ABS-KEY("Gesture observation")
